# Biomechanical Effects of 3D-Printed Bioceramic Scaffolds With Porous Gradient Structures on the Regeneration of Alveolar Bone Defect: A Comprehensive Study

**DOI:** 10.3389/fbioe.2022.882631

**Published:** 2022-05-26

**Authors:** Zhuohui Yang, Chunjuan Wang, Hui Gao, Lurong Jia, Huan Zeng, Liwen Zheng, Chao Wang, Hongmei Zhang, Lizhen Wang, Jinlin Song, Yubo Fan

**Affiliations:** ^1^ Stomatological Hospital of Chongqing Medical University, Chongqing, China; ^2^ Chongqing Key Laboratory of Oral Diseases and Biomedical Sciences, Chongqing, China; ^3^ Chongqing Municipal Key Laboratory of Oral Biomedical Engineering of Higher Education, Chongqing, China; ^4^ Key Laboratory of Biomechanics and Mechanobiology, Ministry of Education, Beijing Advanced Innovation Center for Biomedical Engineering, School of Biological Science and Medical Engineering, School of Engineering Medicine, Beihang University, Beijing, China

**Keywords:** alveolar bone defect, bone scaffold, bioceramic, additive manufacture, biomimetics

## Abstract

In the repair of alveolar bone defect, the microstructure of bone graft scaffolds is pivotal for their biological and biomechanical properties. However, it is currently controversial whether gradient structures perform better in biology and biomechanics than homogeneous structures when considering microstructural design. In this research, bioactive ceramic scaffolds with different porous gradient structures were designed and fabricated by 3D printing technology. Compression test, finite element analysis (FEA) revealed statistically significant differences in the biomechanical properties of three types of scaffolds. The mechanical properties of scaffolds approached the natural cancellous bone, and scaffolds with pore size decreased from the center to the perimeter (GII) had superior mechanical properties among the three groups. While in the simulation of Computational Fluid Dynamics (CFD), scaffolds with pore size increased from the center to the perimeter (GI) possessed the best permeability and largest flow velocity. Scaffolds were cultured *in vitro* with rBMSC or implanted *in vivo* for 4 or 8 weeks. Porous ceramics showed excellent biocompatibility. Results of *in vivo* were analysed by using micro-CT, concentric rings and VG staining. The GI was superior to the other groups with respect to osteogenicity. The Un (uniformed pore size) was slightly inferior to the GII. The concentric rings analysis demonstrated that the new bone in the GI was distributed in the periphery of defect area, whereas the GII was distributed in the center region. This study offers basic strategies and concepts for future design and development of scaffolds for the clinical restoration of alveolar bone defect.

## 1 Introduction

Alveolar bone defect caused by tooth loss, trauma, tumor or infection can seriously compromise individual the quality of life of an individual. As the “gold standard,” autograft and allograft have been widely used in alveolar bone reconstruction surgery (Sheikh et al., 2017). However, limited sources and immunological rejection restrict the use of autograft and allograft tissue replacement (Steffens et al., 2009). Furthermore, current clinical treatments, including bone grafts, guided bone regeneration, distraction osteogenesis, and the use of growth factors/stem cells, are unable to achieve the long-term spatial stability required for osteogenesis (Titsinides et al., 2019). To overcome the above-mentioned inherent drawbacks, more appropriate artificial biomaterials are urgently needed.

Bioceramic materials have become a research hotspot due to their biodegradation, good biocompatibility and osteoconductivity (Du et al., 2018). In particular, HA and β-tricalcium phosphate (β-TCP) and their biphasic composite bioceramic are essential as scaffolds in bone tissue engineering (Kim and Park, 2020). The elements in these materials are similar to those in bone components (Kamalaldin et al., 2019) and have been shown to be effective in clinical indications (Bouler et al., 2017).

Due to the rapid development of additive manufacturing technology (3D printing), the research progress in bioceramics has been greatly promoted. Furthermore, through which the microstructure and composition of biomaterials can be precisely controlled to achieve personalized customization for patients with different clinical needs. Interestingly, inspired by the natural anatomy between bone and cartilage, biomimetic constructs or scaffolds with pore size gradients have been used in bone-cartilage and bone-tendon repair (Du et al., 2017; Zhu et al., 2018; Ansari et al., 2019; Calejo et al., 2019; Tang et al., 2020; Zhang et al., 2020).

However, according to the existing research, in bone defect reconstruction, disputes persist as to whether porous gradient structures are superior to homogeneous structures in terms of osteogenesis and biomechanics and which gradient structure are better. On the one hand, for example, in a comparison study, scaffolds with gradient pore distributions and scaffolds with uniform pore distributions were implanted into equine tuber coxae, gradient scaffolds had less bone remodeling and regeneration (Diloksumpan et al., 2020). In a similar study, porous poly (e-caprolactone) (PCL) and PCL-hydroxyapatite (HA) scaffolds with pore size gradients and ceramic gradients were made by 3D printing, and the results of micro-computed tomography (micro-CT) imaging and uniaxial compression testing showed that the porosity gradient scaffolds deformed much higher than the other groups, while the uniform porosity scaffolds exhibited similar strain values (Bittner et al., 2019). On the other hand, Xiao et al. (2016) prepared bioactive silicate glass scaffolds with uniform and gradient structures, scaffolds with gradient porosity exhibited higher flexural strength and compressive strength than uniform structure in FEA and four-point bending. Li et al. (2019) compared functionally graded structures with uniform structures of additively manufactured (AM) porous iron specimens through experimental and computational analysis and found that topological design with functional gradients controlled fluid flow, medium transport properties and biodegradation behavior.

Therefore, the purpose of the present research is to systematically and comprehensively study the effects of different porous gradient structures on alveolar bone regenerative potential and mechanical properties. Ceramic scaffolds with uniform and two different gradient structures based on the dodecahedron unit cell were designed and 3D-printed *via* digital light processing (DLP)-based additive manufacturing technology. The mechanical properties were evaluated by FEA and compression testing. Cell behavior of the scaffold was assessed *in vitro* with rBMMSCs. Permeability and flow field evaluation in scaffolds with different structures by CFD. Furthermore, osteogenicity was investigated in rat calvarial bone defect and rabbit mandible alveolar defect *in vivo*.

## 2 Materials and Methods

### 2.1 Design and Fabrication of Scaffolds

**FIGURE 1:**
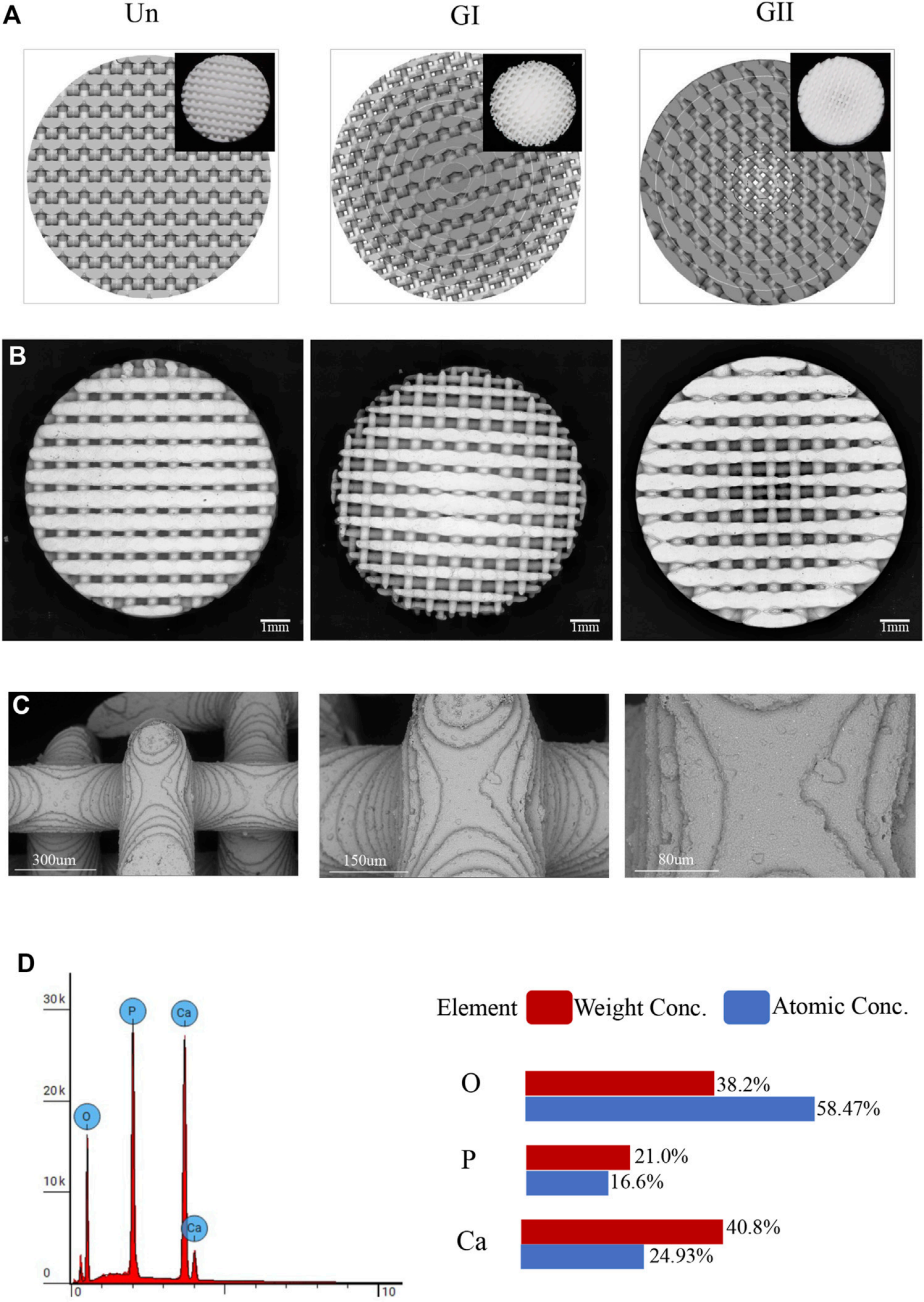
Physical and chemical properties of the scaffold. **(A)**The design of scaffolds structures and the printed images (top right). **(B)** An electro mirror diagram of scaffolds with different structures. **(C)** The images under electroscope of the scaffold are 500×, 1,000×, 2,000×. **(D)** EDS detection of the scaffold.

The porous scaffolds were designed by 3-Matic software (Materialise, Leuven, Belgium) and were divided into three experimental groups: uniform (Un), gradient I (GI), gradient II (GII) (Figure 1A). The pores of the Un group were uniformed and homogeneous 350 μm distributed. The pore size distribution of the GI group gradually decreased from 500 μm in the periphery to 200 μm in the center. The pore size distribution of the GII group was the opposite, the pore size distribution of the GII group gradually increased from 200 μm in the periphery to 500 μm in the center. In the mechanical experiment and FEA, samples with a diameter of 8 mm and a height of 8 mm were used; samples with a diameter of 8 mm and a height of 2 mm were used in the biocompatibility, CFD, and *in vivo* animal experiments. All measured data were measured by Mimics Research software (Materialise, Leuven, Belgium). The designs were exported to STL format.

50 g of bioactive ceramics (7.5 g of HA and 42.5 g of ßTCP powder) (Naton Institute of Medical Technology, Beijing, China) were mixed with 50 g of photosensitive resin to obtain a pre-cursor slurry. Porous scaffolds were fabricated by Additive Printing DLP (AUTOCERA-M, Beijing Ten Dimensions Technology Co. Ltd., China). The exposure time was 3 s for each 30 μm thick slice. The wavelength of the light source was 405 nm. Ceramic samples were obtained after sintering at 1,300°C for 3 h. For *in vitro* and *in vitro* research, scaffolds were treated with autoclave sterilization.

### 2.2 SEM Imaging and Energy Dispersive X-ray Spectrometry Analysis

Three cylindrical scaffolds were selected randomly from each group of as-produced samples and imaged by using a scanning electron microscopy (SEM; Hitachi S-3000N2; Hitachi, Ltd., Japan) (Figures 1B,C). Energy dispersive X-ray spectrometry (EDS, Phenom Prox, Netherlands) was used to determine the distribution of elemental composition and contents of the samples.

### 2.3 Mechanical Evaluations

**FIGURE 2:**
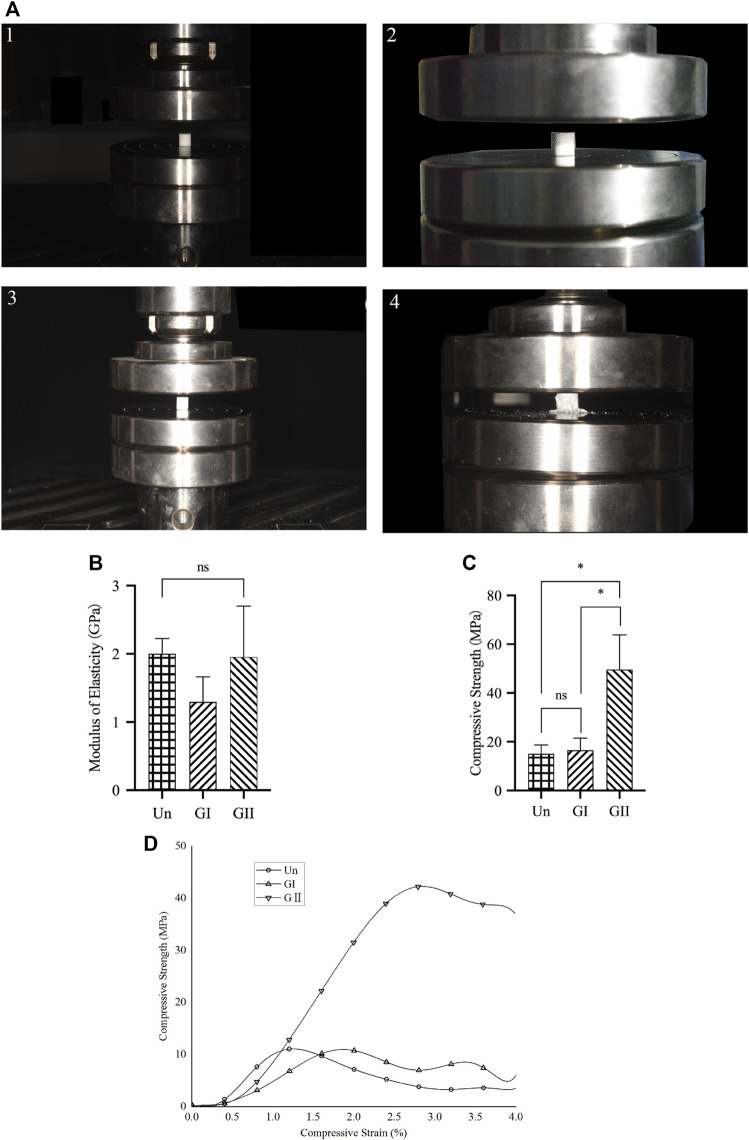
Compression test of porous scaffolds. **(A)** The process of compression testing. **(B)** The elastic modulus of porous scaffolds. **(C)** The compression strength of porous scaffolds. **(D)** Stress strain relationship of porous scaffolds.

**FIGURE 3:**
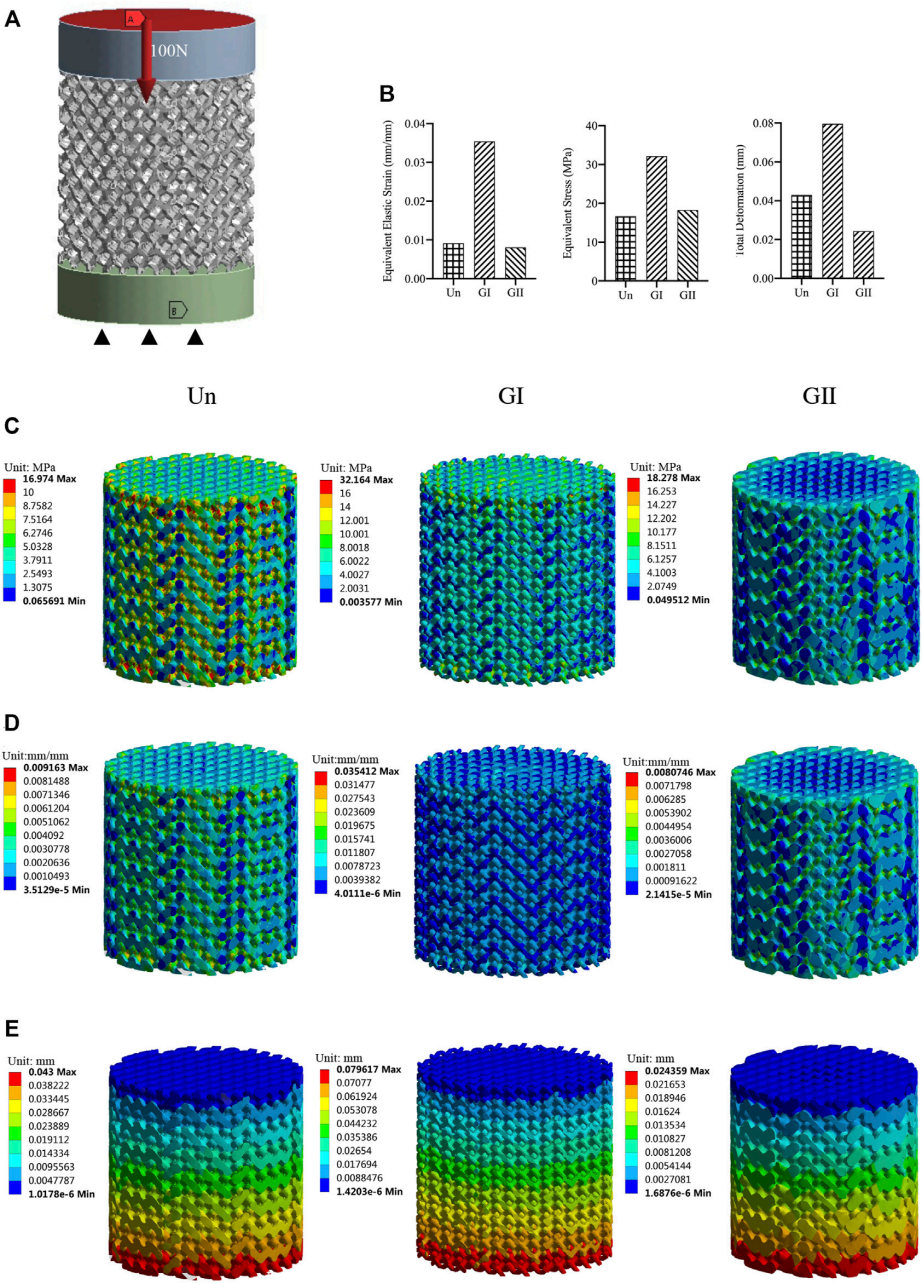
Mechanical properties evaluated by FEA. **(A)** Model with a vertical force 100 N; **(B)** A column chart of stress, strain, displacement. **(C)** Equivalent stress changes with a vertical force of 100 N. **(D)** Equivalent elastic modulus changes with a vertical force 100 N. **(E)** Displacement with a vertical force 100 N.

To evaluate the mechanical properties of the porous scaffolds with different gradient designs, the cylindrical samples (φ:8 mm × 8 mm) from each group were analysed by compression test (Figure 2A) and finite element analysis (FEA) (Figure 3A), respectively. The cylinder specimen was imported into the 3-Matic software, the models were meshed, and the parameters of each model are shown in Table 1, and the body mesh is generated and exported to .cdb format. It is then imported into the ANSYS workbench19.0 software to generate a finite element model. The contact relationship between the scaffold and two plates were set as bonded contact, which meant there was no tangential relative sliding or normal relative separation between the contact surface. The material property parameters [elastic modulus was 1 GPa, Poisson ratio was 0.3 (Ma et al., 2017). The elastic modulus was obtained by compressing a solid bioceramic cylinder with a universal testing machine], the scaffold was fixed, 100 N force was loaded perpendicularly to the porous scaffold at the other end, and the equivalence force and equivalence of the porous ceramic scaffold was analyzed and calculated.

**TABLE 1:** Paramters of FEA and CFD simulation.

Parameters	FEA			CFD		
	Un	GI	GII	Un	GI	GII
Material property	Homogeneous, isotropic, linear elastic (ceramics)			Incompressible and homogenous (tissue fluid)		
Nodes	223231	281063	281025	39890	34128	42006
Elements	742894	1032059	1031907	144048	132181	145742
Mesh Type	Linear Tetrahedron			Linear Tetrahedron		
Average. Aspect Ratio	3.623	3.742	3.741	3.877	3.724	3.904
Average Mesh Size	0.118	0.113	0.100	0.198	0.235	0.169

A porous scaffold was placed in the center of the sub-press plate of a universal test machine (MTS Ltd, China). The instrument was zeroed at an ambient temperature of 25°C, and the vertical force was applied vertically to the scaffold at 1 mm/min under computer control. Continue downward compression until the specimen deformed, and the static compression test results for each sample were used to obtain a stress-strain curve. The elastic modulus was calculated from the linear region of the stress-strain curve. The compressive stress was calculated from the ratio of the actual compressive force to the original cross-sectional area. Strain was determined by calculating the ratio of the percentage deformation of the original spacing segment to the original spacing of the specimen. Three samples for each group were examined.

### 2.4 Computational Fluid Dynamics Simulations

To evaluate the flow field distribution and flow velocity distribution of the three groups, CFD simulation was performed. CAD models (STL format) were imported into ANSYS software (ANSYS, Inc., Canonsburg, PA, United States), and the velocity field was visualized for calculation. We prepared two working conditions: one centered and the other vertical. In the first operating condition, the inlet was from outside to inside, the outlet was from center to top and bottom. In the second operating condition, the direction from inlet to outlet was from top to bottom. The following parameters were selected: the temperature was 21°C and the inlet velocity was 0.1 mm/s. Zero static pressure was defined for the inlet and the outlet with absolute pressure equal to atmospheric pressure, and the steady-state Navier-Stokes equations were applied. No-slip conditions were selected for the walls of the scaffolds, The density (ρ) and dynamic viscosity (μ) of the fluid medium were 1.06 × 10^3^ kg/m^3^ and 3.2 × 10^−3^ Pas, respectively, (Truscello et al., 2012). A series of parameters of the scaffold were used to calculate the effective permeability (k) of Darcy’s law, which is equation:

k=μLQ/A ΔP 

where L represents the height of the scaffold (m), μ represents the dynamic viscosity (Pa s), A represents the area of the cross-section of the scaffold (m^2^), and ΔP represents the pressure gradient (Pa) when the fluid flows through the stent at a rate of Q (m^3^/s).

$$\Delta \text{P}=\;{\text{P}}_{\text{inlet}}-{\text{P}}_{\text{outlet}}$$

Since Darcy’s law is valid for the number of Reynolds number (Re) less than 1, a small flow (Q) is defined for the inlet boundary condition.

Re = ρνd/μ

Where *ν* represents the flow rate and d represents the pore size (m). In this study, small flow (Q) was 5.02 × 10^−9^ m^3^/s, cross-sectional area was 5.02 × 10^−5^ m^2^.

### 2.5 Cell Behavior of Rabbit Mesenchymal Stem Cells on Scaffolds

Samples (φ:8 mm × 2 mm) were used to investigate the cell behavior.

#### 2.5.1 Extraction and Primary Culture of Rabbit Mesenchymal Stem Cells

One percent pentobarbital sodium (Sigma, production batch: 20170318, United States) was injected into the ear vein of each New Zealand rabbit, hair was shaved, and the femur was exposed. A 1.5 cm wound was made in the center of the femur, and the muscle was bluntly dissected until the bone was exposed. Bone needle was inserted, the marrow sheath was pulled out, negative pressure tube was drained, and the sample was rinsed three times with phosphate-buffered saline (PBS) containing 5% double antibody, and transferred to clean bench. The cells were centrifuged at 2,000 r/min for 20 min, and the supernatant was removed. The cells were incubated with F-12 medium containing 1% double antibody and 10% fetal bovine serum at 37°C and 5% CO_2_, with the medium refreshed every 2 days. The third to sixth generation of cells were used in the experiments.

#### 2.5.2 SEM

The scaffold was wetted with culture medium and cells were applied to the scaffold. Rabbit Mesenchymal Stem Cells (rBMSCs) were seeded on the scaffold at 2 × 10^4^, the cells were allowed to adhere after half an hour before an appropriate amount of culture medium was added. On the third and the seventh days of cultivation, the scaffold of the experimental group was removed, rinsed gently with PBS twice, fixed with glutaraldehyde, dehydrated with an ethanol gradient, vacuum freeze-dried, fixed and sprayed, and observed with SEM.

#### 2.5.3 Live/Dead Staining

The staining operation was performed in accordance with the instructions of the Calcein-AM/PI double staining kit (DOJINDO, C542, China). In a 24-well plate, after the scaffold was wetted with culture medium, the cells were seeded at 2 × 10^4^ on the scaffold. After the cells adhesion, more culture medium was added, and live/dead staining was performed after 3 and 7 days. On a clean bench, the medium was discarded, the scaffold was washed twice with PBS and put into a 15 ml centrifuge tube; 10 µL of light-sensitive Calcein-AM stock solution was added. Additionally, 15 µL of PI stock solution was added to 5 ml of PBS solution to make a homogeneous working solution. Under dark conditions, 1 ml of PBS solution and 500 µL of dye solution were added to each well, incubated for 15 min in the incubator, and images were collected under a fluorescence confocal microscope (Leica, TCS. SP8, Germany).

#### 2.5.4 CCK-8

When four groups of cells (including blank control group) were cultured on the scaffolds (2 × 10^4^ cells) in 24-well plates for 1, 3 and 7 days, the effect of scaffolds on cell viability was tested by the CCK-8 method. Nine multiple wells were set up in each group and cultured until the detection time. Next, the ratio of CCK-8 working solution (Beyotime, C0038, China) to medium was 10:1 and initialized in the incubator for 2 h. Then, 100 μL of working solution was taken from each well and transferred to a 96-well plate, and the absorbance was detected at 450 nm with a microplate reader (ELX800, Bio-Tek, United States).

### 2.6 Animal Studies

In order to explore the effects and differences of different gradient structures on osteogenesis *in vivo*, the classic bone defect model—the rat calvarial defect model was made. Further, in order to be close to the clinical situation, we made alveolar bone defect of rabbit. The animals were obtained from the experimental animal center of Chongqing Medical University. All experimental animals were maintained in the animal facility of the experimental animal center of the Chongqing Municipal Key Laboratory of Oral Biomedical Engineering of Higher Education, and the experimental protocol was reviewed and approved by the Research Ethics Committee of College of Stomatology, Chongqing Medical University, Chongqing, China [CQHS-REC-2021 (LSNo.54)]. All procedures were strictly carried out in accordance with the approved guidelines. Twenty-four male Sprague-Dawley (SD) rats (200 ± 50 g, 8 weeks old, Animal Center of Chongqing Medical University) and 24 male New Zealand rabbits (3.0 ± 0.2 kg, Animal Center of Chongqing Medical University) were acclimatized to the laboratory for 14 days prior to surgery and randomly assigned to three groups. The SD rats were anesthetized by isoflurane inhalation (Hebei Yipin Pharmaceutical Co., Ltd., China) (1.5 L/min), the calvarial bone was shaved and sterilized, 1.5 cm incision was made in the center of the calvarial bone (Supplementary Figure 1A), the skin and muscles were peeled off to expose the surface of the bone. Cylindrical implant holes with a diameter of 8 mm and a height of 2 mm were prepared by using a saline-cooled dental implant system. After the bone pieces were removed, saline was used to flush the hole.

The rabbits were anesthetized *via* ear vein injection with 1% pentobarbital sodium (3 ml/kg), the left mandible of each rabbit was shaved and sterilized, 1.5 cm incision was made at the lower edge of the mandible, and the muscles were separated and peeled off the fascia to expose the bone surface (Supplementary Figure 1B). A cylindrical implant hole of 8 mm diameter and 2 mm depth was prepared in the mandible alveolar bone by using a tooth implantation system under cooling with saline. After the bone pieces were removed from the jawbone of a rabbit, the mandible molar can be seen (Shah et al., 2016).

Samples (φ:8 mm × 2 mm) were implanted into skull of rats or mandible alveolar bone of rabbits, and the animals received an intramuscular injection of penicillin (4,000 U/rat, 40,000 U/rabbit) after surgery. After 4 and 8 weeks of the operation, the animals were sacrificed in a euthanasia box. The entire calvarial bone and mandible alveolar bone were removed and analysed with micro-CT scanning (SCANCO VivaCT40, Switzerland); the scanning data of the samples were used to reconstruct 3D images in Mimics Research 19.0 Software (Figure 6A), and the volume fraction of bone to tissue volume (BV/TV) was calculated. The gray values were extracted for density distribution analysis (Figures 6B1,C1). Afterwards, we made 8 concentric rings with a radius of 0.5, 1, 1.5, 2.0, 2.5, 3.0, 3.5, and 4 cm by 3-Matic software (Materialise, Leuven, Belgium), the width of each concentric ring was 0.5 cm and was obtained by a Boolean operation (Figure 7A1). The images of the new bone were imported into the software in STL format (Figure 7A2). Boolean intersection was performed between the new bone and concentric rings (Figure 7A3), and the new bone volume in each ring was obtained (Figure 7A4).

### 2.7 Histology

Samples were fixed in 4% paraformaldehyde, gradient dehydrated with alcohol, and embedded in methacrylate (Sigma, United States). Samples were sectioned by a diamond cutting system (EXAKT, E300 CP/400CS, Germany). To observe the osteogenesis inside the cross-sectional scaffold, we chose the cross-sectional section that the thickness of the section was approximately 20 µm. Then, the sections were stained with Stevennell’s blue and Van Giesen’s picrofuchsin. Images were scanned using a digital slice instrument (OLYMUS, VS.200, Japan). Semiquantitative analysis and statistics were performed by Image Pro Plus 6.0 software (Media Cybernetic, United States). The bone ingrowth area was the ratio of the new bone tissue area of the porous scaffold to the total pore area in the scaffold; the new bone ingrowth depth was the length of the new bone ingrowth from the center of the scaffold. Three different sections were examined for each group.

## 3 Results

### 3.1 Scaffold Characterization

SEM imaging (Figure 1C) showed the surface morphologies of the scaffolds, the surface structures of the bioceramic scaffolds were similar to each other, especially layer-by-layer fabrication can be seen at the scaffold junction. Through elemental analysis, it can be seen that the scaffolds were composed of calcium, phosphate, and oxygen (Figure 1D), which contented 38.2%, 21.0%, and 40.8% by weight, respectively. Atomic contents were 58.47%, 16.6%, and 24.93%, respectively.

### 3.2 Mechanical Characterization of Porous Scaffolds

The compression test showed that there was no significant difference in the elastic modulus of the three groups (Figure 2B), and the GII scaffolds had better compressive strength than the other groups (*p* < 0.05) (Figure 2C), as illustrated by stress strain graphs (Figure 2D). Finite element analysis (FEA) was carried out by using the Ansys Workbench program (Ansys, United States) under the following boundary conditions: fixation for the bottom surface was fixed, and then a force of 100 N was applied at the surface (Figure 3A). The data showed that the peak equivalent stress of the Un (16.973 MPa) and the GII (18.278 MPa) were lower, the equivalent elastic strain was smaller, the total displacement after being subjected to pressure of the GII was the smallest, and there was no obvious stress concentration area. The stress concentration of the Un was significant and distributed in the cross section. Likewise, the equivalent elastic strains of the Un (0.009163 mm/mm) and the GII (0.0080746 mm/mm) were smaller than that of the GI (0.035412 mm/mm), and the displacement of the GII (0.024359 mm) was smaller than that of the Un (0.043000 mm) and the GI (0.079617 mm). Then we continued to verify its mechanical properties through compression tests, and the results were consistent with FEA results. There was no significant difference between the elastic modulus of the Un and the GII, and the GI was lowest among the three groups. However, the compressive strength and peak stress of the GII group were significantly higher than those of the other two groups.

### 3.3 Computational Fluid Dynamics Analysis

**FIGURE 4:**
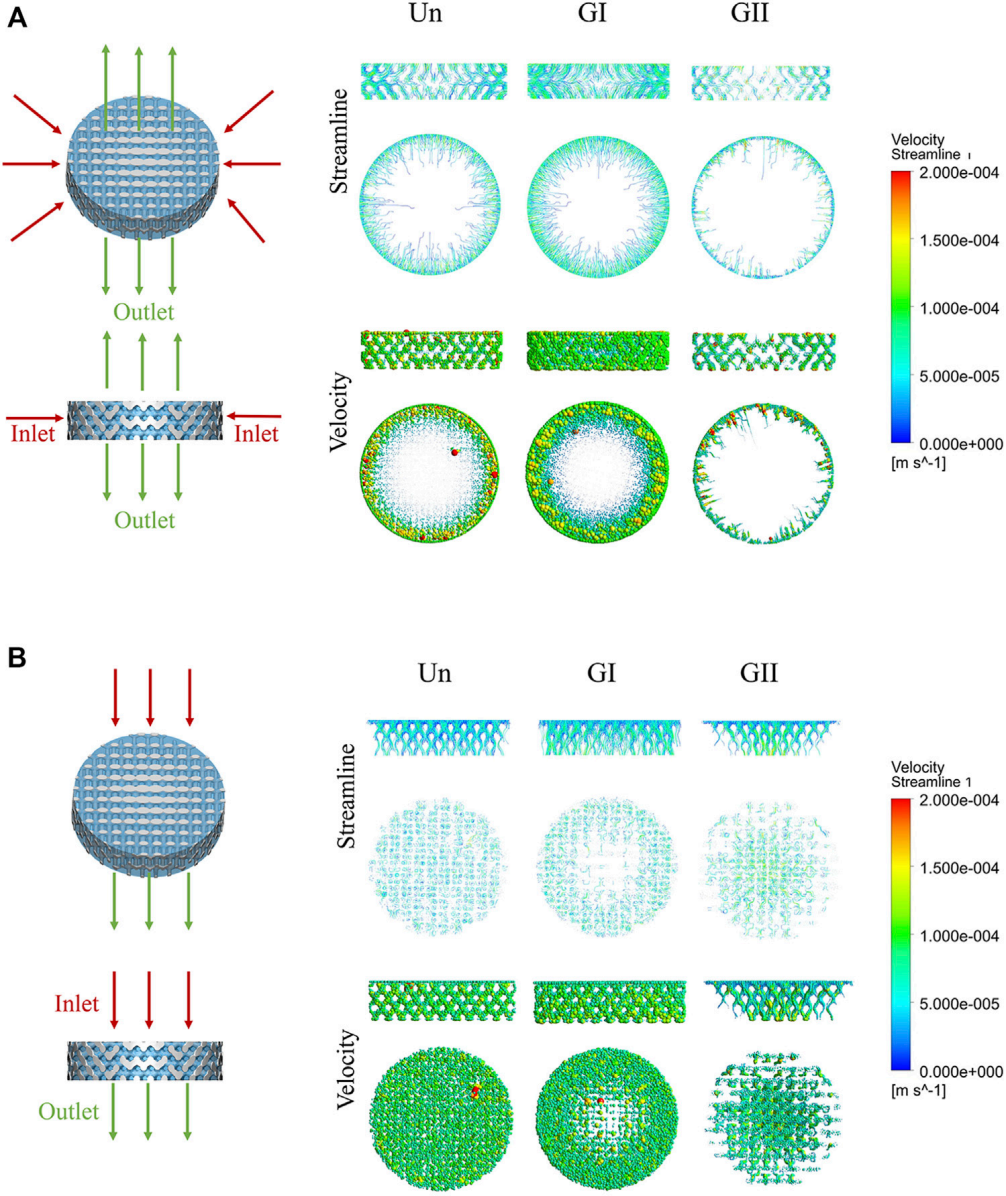
CFD simulation analysis of porous scaffolds with different structures. **(A)** Horizonal direction of CFD simulation, the inlet was from outside to inside, the outlet was from center to top and bottom. **(B)** Vertical direction of CFD simulation, the direction from inlet to outlet was from top to bottom. Streamline and velocity field distribution and variation from the inlet to the outlet.

The CFD model is shown in Figures 4A,B. Results of CFD analysis were shown in Table 2 **.** The fluid phase was considered to be tissue fluid, after the boundary conditions were applied. The velocity field represented the velocity changes, and the streamline field represented the velocity direction of the fluid from the inlet to the outlet. The degree of redaction of the streamline cluster reflects the difference in velocity in the flow field at that time. The streamline field and velocity field decreased gradually from the inlet to the outlet side in the three groups. Under simulation condition of horizontal direction, the three groups of flow field motion were distributed on the periphery of the scaffold, and fluid of the GII has the smallest range of motion while the GI had widest range of motion. Through the calculation, the results showed that the overall effective permeability of the GI group (1.07E + 05 mD) was more than 50 times higher than that of the Un (2.05E + 03 mD) and GII groups (7.30E + 02 mD), which was calculated as shown in Table 2. Under simulation condition of vertical direction, the fluid movement of the Un was evenly distributed across the entire scaffold, and the fluid velocity was more uniform. The fluid motion of the GI group was mainly distributed in the periphery of the scaffold, and the central flow rate was significantly lower than that of the periphery. The GII group was the complete opposite, that was, the fluid motion was mainly distributed in the center of the scaffold, and the central flow rate decreased to the periphery of the scaffold. In the three groups, the effective permeeabilith of the GI (7.13E + 04 mD) was more than 10 times higher than that of Un (6.71E + 03 mD) and GII (5.19E + 03 mD). Under two kinds of operations, the Reynolds number of the three groups were less than 1, which was in range of application of Darcy’s law. The simulation results showed that the GI group had the widest distribution of flow fields and the strongest permeability in the three groups.

**TABLE 2:** Results of CFD analysis.

Parameters	Horizontal Direction			Vertical Direction		
	Un	GI	GII	Un	GI	GII
Gradient Pressure (Pa)	3.14E-01	6.06E-03	8.87E-01	9.65E-02	9.09E-03	1.25E-01
Reynolds Number	4.11E-03	5.08E-04	1.61E-03	5.68E-03	4.13E-03	7.74E-03
Effective Permeability (mD)	2.05E + 03	1.07E + 05	7.30E + 02	6.71E + 03	7.13E + 04	5.19E + 03

### 3.4 Cell Adhesion, Viability and Differentiation on Porous Scaffolds

**FIGURE 5:**
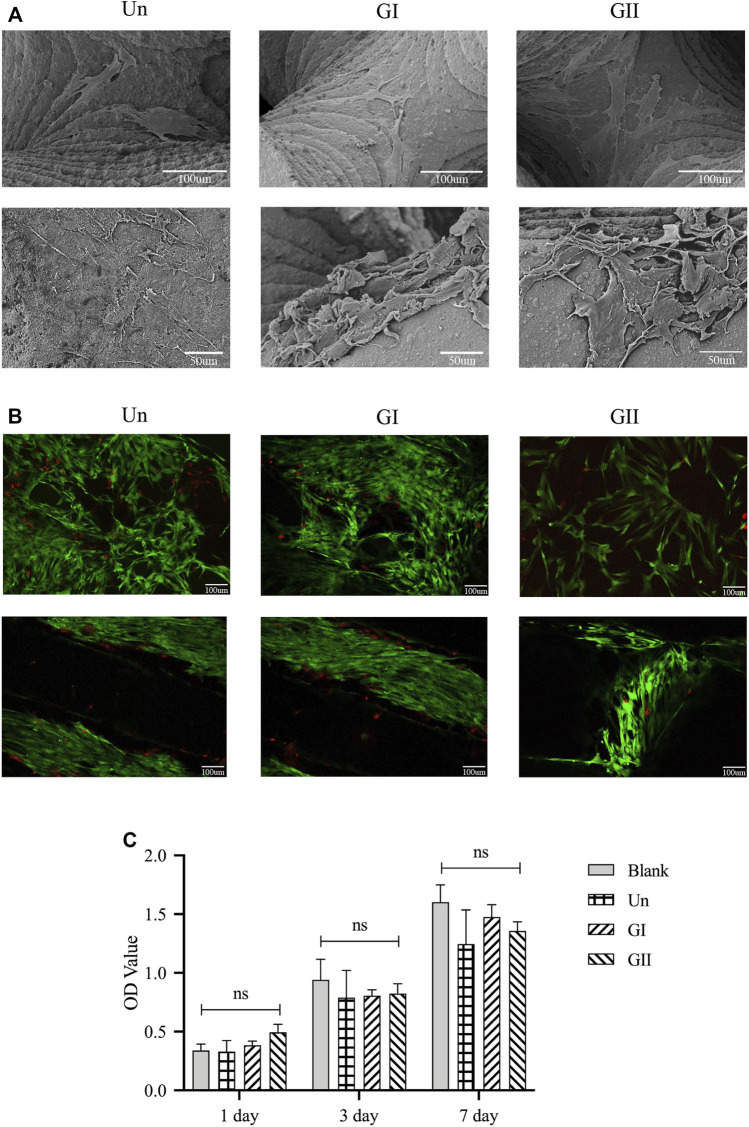
Cell adhesion, growth, morphology and viability on the porous scaffolds. **(A)** SEM images of rBMMSCs morphology on scaffolds after being cultured for 3 and 7 days; **(B)** Live/Dead staining of rBMMSCs on the scaffolds after 3 and 7 days of culture, green represents living cells, red represents dead cells. **(C)** rBMMSCs adhesion and proliferation on the scaffolds.

SEM images showed the rBMSCs morphology on the porous scaffolds after 3 and 7 days of culture growth (Figure 5A). The cells adhered and proliferated on the surface of the scaffolds. The cell viability on the porous scaffolds was evaluated by live/dead staining after 3 and 7 days of culture growth. Fewer dead cells (stained red) were observed on the scaffolds. Overall, cells survived on all scaffolds and grew widely, all over the field of view. All of images indicated that all three scaffolds had good cytocompatibility *in vitro* (Figure 5B). The CCK8 (Figure 5C) demonstrated that there was no significant difference comparing with the blank group at the 1, 3, and 7 days. Cell proliferation was positively correlated with time, as the OD values of all groups increased with time. There was no significant difference among the four groups, which indicated that the material had good cytocompatibility, and different gradient structures had no significant effect on cell proliferation *in vitro* (*p* > 0.05).

### 3.5 Micro-CT Analysis

**FIGURE 6:**
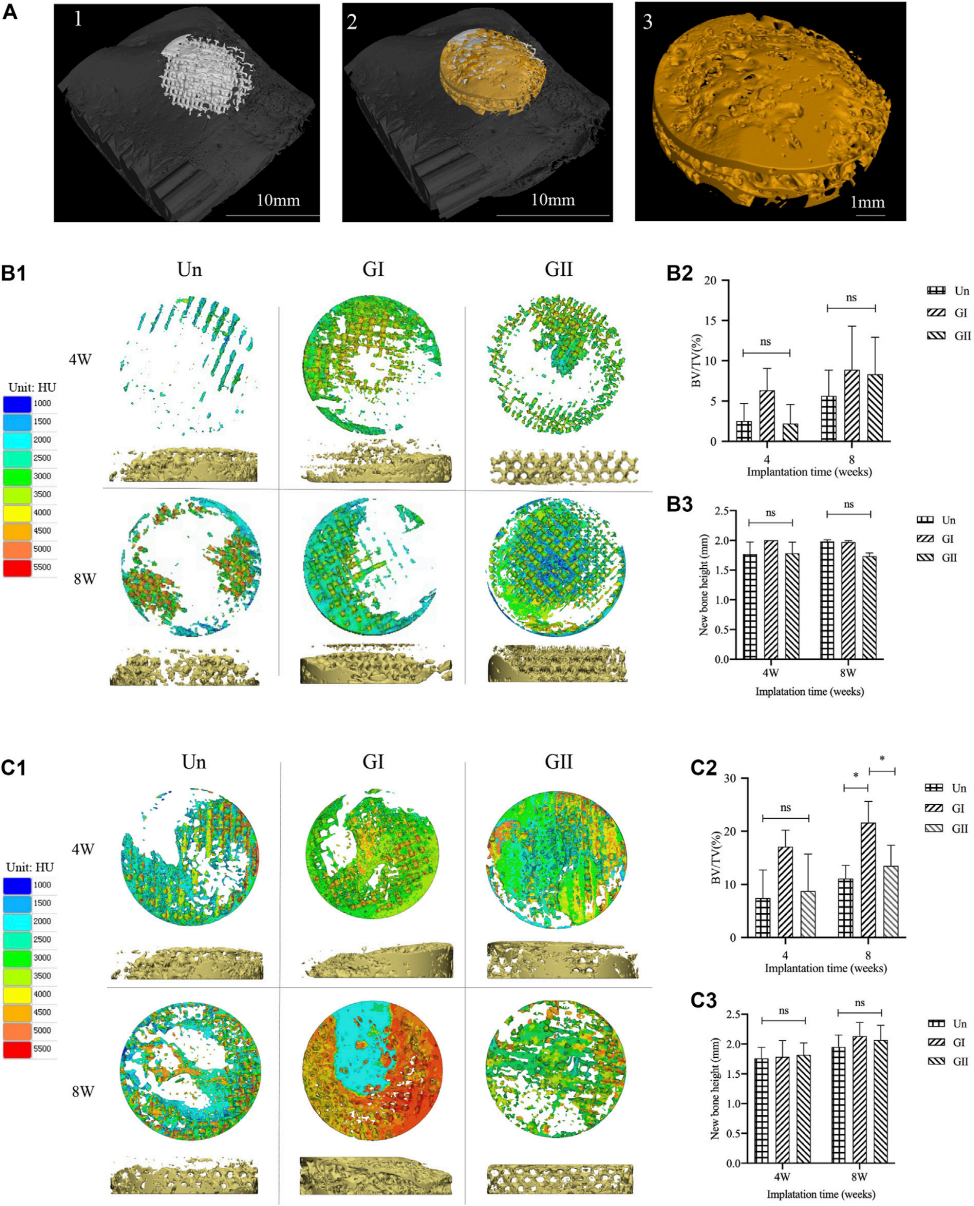
Micro-CT analysis of animal experiments. **(A)** The new bone was extracted. **(B1)** The density distribution of new bone in the rats. **(B2)** BV/TV of rats. **(B3)** The height of the new bone in rats. **(C1)** The density distribution varies of new bone in the rabbits. **(C2)** BV/TV of rabbits. **(C3)** The height of the new bone in rabbits.

All animals received the anesthetic injection and surgery well, there was no infection or inflammation at the wound after the operation, and the scaffolds were stable for 4 and 8 weeks. To validate whether the gradient group was better than the uniform group, the Un group served as the control group, and micro-CT scans were used to analyse the samples, Mimics and 3-Matic software were used to analyse new bone density (Figures 6B1,C1), and the density distribution was converted to an image based on the grayscale value of the new bone. Images were reconstructed by using VG Studio MAX software (Volume Graphics, Germany). The results of bone tissue volume/total tissue volume (BV/TV) (the right part of Figures 6B2,C2) showed that whether at 4 or 8 weeks, the GI group generated more new bone than the other groups with or without significant differences which indicated that there was persistent bone tissue ingrowth in the GI. Meanwhile, the new bone density was characterized by not only the density of the new bone but also visual observations of the distribution of the new bone. There were some differences in new bone distribution of rats and rabbits. In rats, new bone in both the Un and the GI was more concentrated in the periphery. While in rabbits, the Un was more discretely distributed, and the GI was more widely distributed throughout the defect, especially in the center of the defect. However, the GII group showed extensive growth of bone growth in the center of the scaffold at 4 or 8 weeks regardless of rats or rabbits, indicating that the newly formed bone tissue in the GII was preferentially distributed in the center. The results for new bone height (Figures 6B3,C3) showed that there was not statistically significant difference among the three groups. However, the bone formation height of the GI was slightly higher than that of the other groups, which demonstrated that the GI promoted vertical bone regeneration more than the other groups.

### 3.6 Concentric Rings Analysis

**FIGURE 7:**
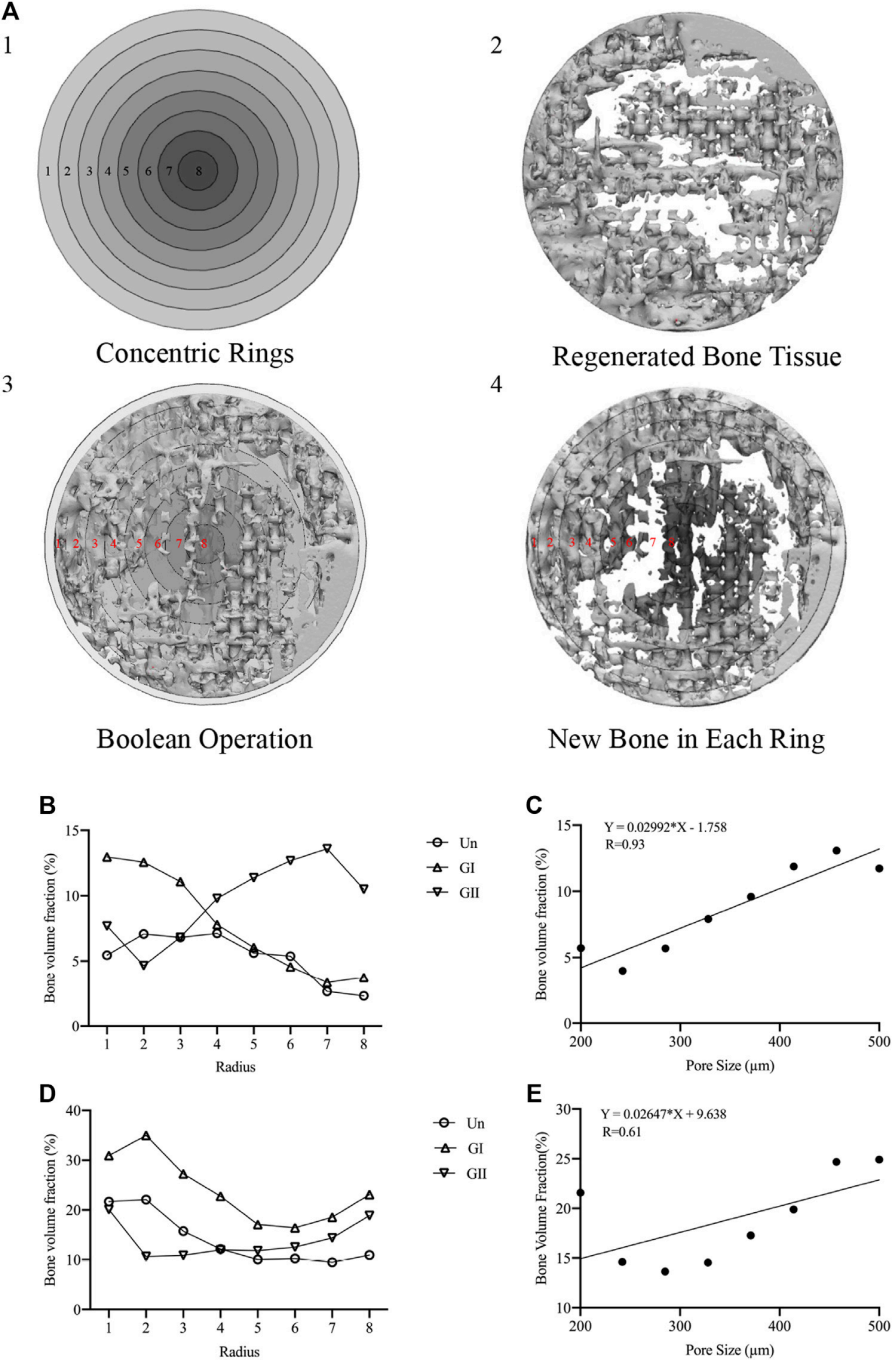
Concentric rings analysis. **(A-1)** Concentric rings designed by 3-Matic software. **(A-2)** regenerated bone in STL format. **(A-3)** Boolean operations of A1 and A2. **(A-4)** New bone in each bone was obtained. **(B)** New bone of the 8 rings of in rats. **(C)** Linear regression plots to depict the relationship between pore size and new bone volume in rats. **(D)** New bone of the 8 rings of in rabbits. **(E)** Linear regression plots to depict the relationship between pore size and new bone volume in rabbits.

Since the total bone mass provided only a general quantitative analysis, comparing the amount of new bone in each concentric ring allows for a more intuitive and precise quantification of the new bone distribution than total bone mass. Thus, we made 8 concentric rings to analyse the distribution of new bone. The results implied that the outer ring always showed the most bone volume, and the bone volume of the GI group was larger than that of the other groups, especially in the outer rings (rings 1–3) (Figures 7B,D). More than that, the total amount of new bone decreased from the periphery to the center. The GII exhibited the opposite trend, that was, the inner rings (rings 7–8) performed better than the outer rings, but the result was not obvious. On the whole, there was not significant difference in rings 4–6. These results suggested that comparing with the Un, the GI mainly promoted peripheral bone formation, while the GII mainly accelerated central bone regeneration of the defect area. Moreover, we did a linear regression analysis of new bone mass and pore size, and found that although the slope of the linear relationship varies between rats and rabbits, as the pore size increased, new bone production increased in the GI group and GII group, rats (Figure 7C) and rabbits (Figure 7E). This may be due to the physiological loading of the stent in the alveolar bone, but it needs to be further explored.

### 3.7 Histological Evaluations

**FIGURE 8:**
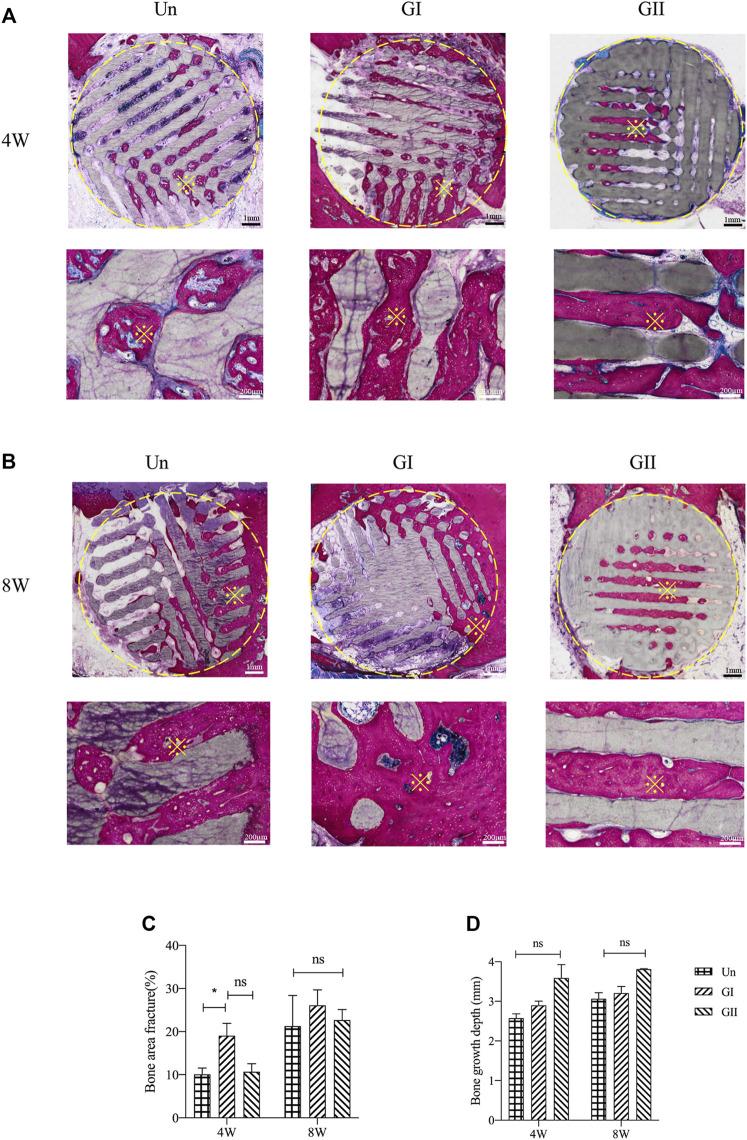
Histological analysis of porous scaffolds implanted into rats after 4 weeks **(A)** and 8 weeks **(B)**, below is a partial enlargement of the figure above, ※with yellow represents locally magnified point. Bone area fraction **(C)** and bone growth depth from the outer of the scaffold into the center **(D)**.

**FIGURE 9:**
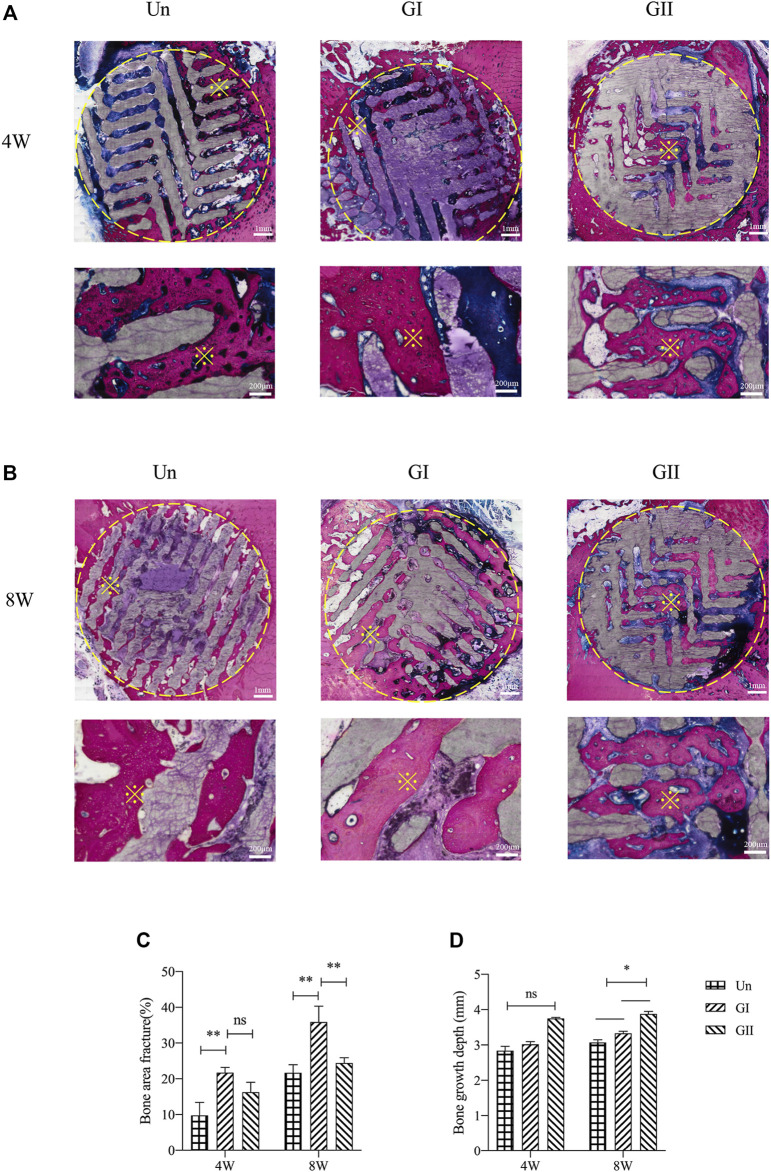
Histological analysis of porous scaffolds implanted into rabbits after 4 weeks **(A)** and 8 weeks **(B)**, below is a partial enlargement of the figure above, ※with yellow represents locally magnified point. Bone area fraction **(C)** and bone growth depth from the outer of the scaffold into the center **(D)**.

Histologically images of SD rats (Figure 8) and New Zealand rabbits (Figure 9) at 4 and 8 weeks of ages were obtained through OlyVIA 3.1 (OLYMUS, Japan) software, in which red represents the new bone, and blue represents newborn collagen fibers in the scaffolds. The images demonstrated that the GI generated more bone than the other groups, and the new bone grew deeper in the GII scaffolds than the other groups. The new bone was more peripherally located in the GI group, whereas in the GII group, the new bone was located in the center. The distribution of in the Un group was scattered.

### 3.8 Statistical Analysis

The experimental data were expressed as mean ± standard deviation (Mean ± S.D.), one-way ANOVA was used for the intergroup comparison, Tukey method was used for the two-two comparison, and *p* < 0.05 was considered to be statistically significant (*p < 0.05, **p < 0.01).

## 4 Discussion

In this study, we systematically and comprehensively studied the effects of gradient structure on biomechanics, fluid mechanics, biocompatibility, and osteogenesis. Our findings suggested that the nutrient/waste exchange of the gradient structure inside the scaffold facilitated the widespread distribution of cells and nutrients. In particular, mediator exchange inside the scaffold played an active role in bone regeneration. The center of scaffolds exhibited poor osteogenicity due to a lack of nutrients and blood flow. Whereas, new bone formation of the GII usually occurred internally. This may be explained by the GII delivering oxygen and nutrients to cells in the center of the scaffold through the blood, which subsequently stimulated bone differentiation.

The microstructure of bone scaffolds regulates the fate of differential cell and tissue (Swanson et al., 2021) and plays a decisive role in supporting new bone growth and vascularization in clinical repair of defect (Roseti et al., 2017; Luo et al., 2022). According to previous study, it influences molecular diffusion and oxygen diffusion, and regulates vascular invasion and bone ingrowth (Cavo and Scaglione, 2016). With the development of bionics, many scholars have not only simulated the shape and function of natural cancellous bone, but also studied the heterogeneous microarchitecture within the scaffolds (Wu et al., 2017).

Recently, it is generally accepted that the type of unit cell, pore porosity and pore size are key components of microstructure, and some progress has been made in the studies of how these factors influenced bone regeneration. First, there are many studies about the type of unit cell. For example, Zadpoor et al. (Kolken et al., 2021) assessed the mechanical properties of six different unit cells of AM architectural materials and found that the diamond, body-centered cubic, and rhombic dodecahedron lattices showed ideal properties. Gao et al. (2019) evaluated four configurations to reconstruct mandible defect and found that an optimized implant was constructed with a regular dodecahedron unit cell. Wang et al. (2018) evaluated the effect of porous Ti with different pore structures on bone integration and bone formation and observed that the diamond crystal lattice and tetrahedral structure provided good osteointegration and osteogenesis. Second, through linear, power-law and exponential reforming, it was found that the optimal pore microarchitecture of Ti scaffolds for cortical bone was <212 µm with volumetric porosity values of 27–37%, and the optimum pore microarchitecture for trabecular bone was in the 300–500 µm range with volumetric porosity values of 54–58% (Torres-Sanchez et al., 2017). Similarly, porous tantalum (Ta) scaffolds were constructed with porosity of 25%, 55%, 75%, and 85%, respectively. It was found that the porous Ta scaffolds with pore diameters of 400–600 μm and porosity of 75% were beneficial to osteogenesis and osteointegration (Luo et al., 2021). Third, the variation of porous size is another key issue in the design of the scaffolds (Lee et al., 2014; Li et al., 2016; Ghayor and Weber, 2018; Wu et al., 2021). There are at least two basic types of porous size gradients. In one type, pore size decreased from the center to the perimeter enhances the mechanical performance, improves the transport of nutrients and oxygen to the deepest cells (Xiang-Yu Zhang et al., 2019), and promotes cell adhesion and osteogenic differentiation (Di Luca et al., 2016). The core of the scaffold has a larger pore size for nutrient supply and newly formed bone. In the other type, pore size increased from the center to the perimeter promotes the initial growth of bone and blood vessels to grow into the scaffolds, and facilitates blood and nutrient diffusion to the center. Herein, this study comprehensively compares the gradient structures with the homogeneous structure and then compares the two gradient structures.

Various methods, including electrospinning, freeze-drying, solvent casting/salt leaching and gas-based techniques, have been used to generate porous scaffolds (Annabi et al., 2010). The disadvantages of these techniques include uneven and inaccurate scaffold porosity, which cannot achieve precise pore sizes and pore interconnectivity (Dehghani and Annabi, 2011). In particular, extrusion-based bioprinting has been widely used in most existing commercial bioprinters (Lei Zhang et al., 2019), but limited resolution of the printing makes modeling of the scaffold less accurate. DLP-based stereolithography as an advanced technology can ensure that the print-based model is consistent with the preparation, and the error can be as small as 100 µm (Wubneh et al., 2018; Schmidleithner et al., 2019). The present study considered that a porous size (<200 µm) that is too small to print and process, a porous size (>500 µm) that is too large affect the mechanical strength of the scaffold. Thus, the variation range of pore size was set as 200–500 µm.

For bioactive scaffolds, it is of great significance to possess the appropriate biomechanical properties to adapt to natural bones (Turnbull et al., 2018), excessive mechanical properties will lead to stress shielding (Sola et al., 2016), while too small mechanical properties cannot maintain the space maintenance of scaffolds, resulting in bone resorption (Kadkhodapour et al., 2015; Vaquette et al., 2021). Moreover, biomechanical properties also influence cell behaviors, modulate local environment (Mokhtari-Jafari et al., 2020). According to the FEA and compression tests in this study, although the compressive strength and peak stress of the GII were significantly higher than those of the other two groups, the elastic modulus of the three groups were all close to those of cancellous bone and cortical bone (0.5–20 GPa) (Parthasarathy et al., 2010). Our results demonstrated that the mechanical properties of bioactive ceramics were adapted to human natural bone.

Next, biocompatibility of scaffolds was observed, and our results indicated that there were no significant differences in cell adhesion and proliferation on scaffolds of different structures. Furthermore, the differences in permeability between different groups were analysed by simulation. The biological manifestations of porous scaffolds are closely related to the nutritional diffusion and metabolism inside the scaffolds. Similar to the findings by Mokhtari-Jafari et al. (2020), computational modeling and multiscale systems biology elucidating the contributions of endothelial cell proliferation and migration. The simulated results suggested that the permeability of the GI group (pore size increases from the center to the perimeter) was better than that of the other two groups under any conditions, which was consistent with the *in vivo* test results. This study also indicated that as a technical means, CFD might be able to predict differences in blood vessel growth and even bone growth within scaffolds of different architectures.

According to the results of *in vivo* experiments, the new bone in the GI far exceeded the other two groups, and the GI may affect osteogenesis by influencing fluid flow in a wider range inside the scaffold, which was consistent with the CFD results. The reason may attributed that large pores at the periphery of the scaffold mediated new bone ingrowth. The GII performed slightly better than the Un group, perhaps because the GII accelerated central bone regeneration more than the Un. Not only that, the distribution of new bone was also consistent with concentric rings analysis, the new bone of the GII was distributed in the center of the defect area, implying the GII induced deeper bone ingrowth, which might be explained by the stronger media and blood flow in the center of the scaffold. In addition, new bone formation was clearly observed in histological evaluations, and its quantitative analysis was consistent with micro-CT results. Interestingly, we found that the height of new bone in the rabbit defect model exceeded 2 mm, especially in the GI, implying that the scaffolds had an excellent ability to guide bone regeneration, which may mean that bone augmentation can be achieved by controlling the gradient structure design.

The limitation of our work should be discussed herein. The gradient changes were limited to two-dimensional cross sections of surfaces and did not extend to three dimensions. The gradient changes were linear relationships; non-linear and bilinear changes were not taken into account in this study. Basic trends were discovered in this research, and the gradient structures need to be optimized further in the future work.

## 5 Conclusion

In this study, we designed, fabricated and evaluated the performance of bioactive ceramic porous scaffolds with different microstructures for alveolar bone defect. It was found that the porous gradient distribution was more conducive to osteogenesis and maintained better mechanical properties than the uniform distribution. The larger pore size (400–500 µm) is near the host bone, the better tissue permeability and bone conductivity of the scaffolds. The smaller pore size (200–300 µm) is near the host bone, the better mechanical properties of the scaffolds. The results of this study can help us further optimize the structural design of bioceramic scaffolds to obtain the excellent biomechanical properties and better osteogenic properties, which will greatly advance the clinical translation of bioceramics in alveolar bone defect reconstruction.

## Data Availability

The original contributions presented in the study are included in the article/Supplementary Material, further inquiries can be directed to the corresponding authors.
