# Supplementary material for: Biomechanical Effects of 3D-Printed Bioceramic Scaffolds With Porous Gradient Structures on the Regeneration of Alveolar Bone Defect: A Comprehensive Study
Source: Front Bioeng Biotechnol. 2022 May 26;10:882631. doi: 10.3389/fbioe.2022.882631 (PMC9177945; doi:10.3389/fbioe.2022.882631)
Supplement: Supplementary file 1 [file DataSheet1.DOCX]

Supplementary Material

# Supplementary Figures

## Supplementary Figures


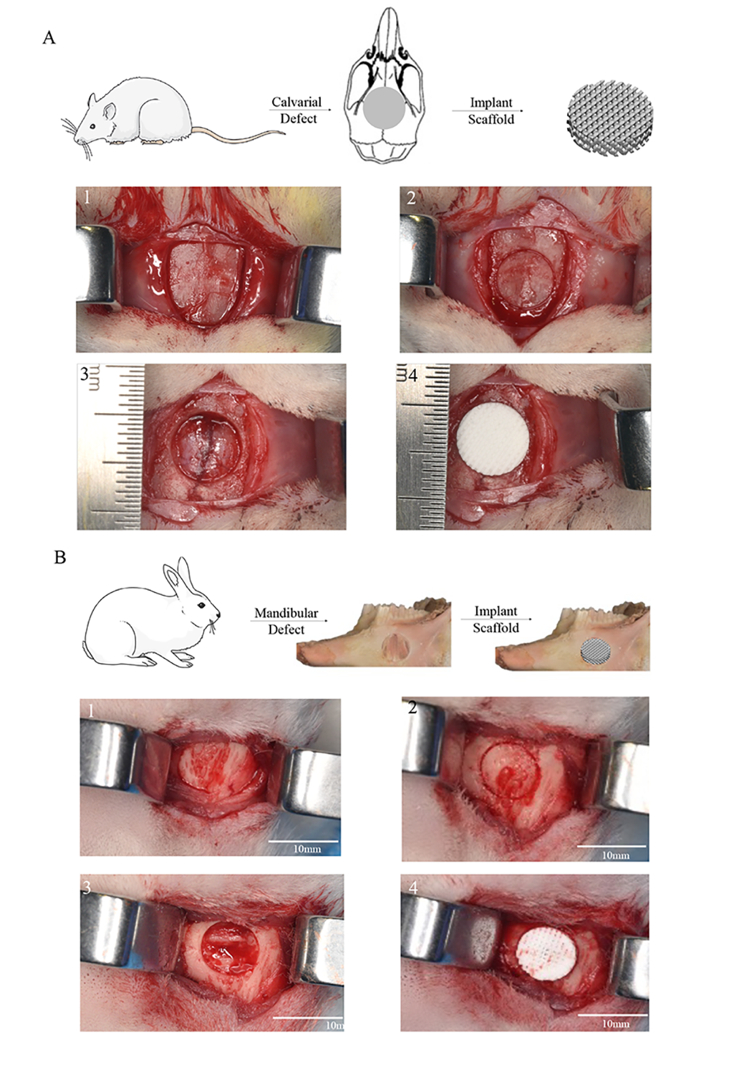


**Supplementary Figure 1.** Animal experiments in scaffolds implantation. The surgical procedure for implantation into the rat calvarial bone(A). and the rabbit mandible alveolar bone(B).
